# "The Hospital" and the Present Position

**Published:** 1918-12-07

**Authors:** 


### A STATE MEDICAL SERVICE.

### "THE HOSPITAL" AND THE PRESENT POSITION.

(BY A CORRESPONDENT.)

EVERYONE who is interested in the future of the medical profession and is anxious to secure for the community improved medical guidance and aid must feel indebted to Colonel Maurice for the articles on a State Medical Service which have appeared in recent issues of THE HOSPITAL. The term has been frequently quoted, but without any precise definition. Colonel Maurice has supplied it with an exact meaning, and his exposition must have involved a considerable expenditure of time and thought. He has worked out both principles and details, and has presented his plan in a lucid and engaging fashion. Once grant the position that it is in the interests of the nation that the State should organise the medical profession as a branch of the Civil Service and here is a plan by which the proposal could be made actual. Hence for those who accept this position the scheme submitted may reasonably be made a basis for discussion and for constructive criticism. But what of those who are not convinced of the soundness of the general principle? They are surely entitled to their opinion and to the expression of it. For the holders of such an opinion it would be an idle task to enter upon "constructive criticism" of the details of a scheme to the central principle of which they are entirely opposed. To imply, as does THE HOSPITAL of November 30 (p. 178), that persons who adopt this attitude are deficient in "statesmanship" is to ask that opponents of the "second reading" of the scheme shall spend their time on a discussion "in committee." It is only when the "second reading" is carried that such discussions become appropriate. Hence while Colonel Maurice's proposals may reasonably claim "constructive criticism" from those who accept a State Medical Service as advisable or inevitable, no such demand can properly be addressed to others who contest this central proposition. To reproach these latter with a failure to supply criticism of the variety invited is unwarranted, seeing that according to their view what is required is not improvement of the details of the scheme, but a rejection of its central principle. Censure so phrased is neither "statesmanship" nor "practical politics."

Colonel Maurice himself has told us "I have not advocated State Medical Service" (*British Medical Journal*, November 30, p. 616). His object, therefore, is to put into a practical form the ideas of those who do pursue such advocacy, and he quite reasonably asks from them constructive criticism. He at all events will not join in condemning as deficient in "statesmanship" those who are silent about details intended to secure the application of a principle which their judgment opposes. His purpose plainly is to favour discussion among those who accept the central doctrine of a State Medical Service and not to engage others to whom this central doctrine is anathema. The latter need only appear when the principle of such a service has been accepted.

With all that is said of the admirable qualities of Colonel Maurice's atricle in THE HOSPITAL of November 30 (p. 178) all will agree, and the supporters of such a system are to be congratulated on so precise and practical an exposition of their ideas. Never have they had so good a chance for "constructive criticism," and their endeavours in this direction are awaited with interest. Others in the meantime may be silent until by the provision of such criticism the scheme has taken the form which its sympathisers desire. To interfere now would be premature and even presumptuous, for every comment would be met by the rejoinder that its object was the destruction of the entire scheme. This, indeed, is the object of those who believe that a State Medical Service is opposed to the interests both of the medical profession and of the public. The way to attain this object, however, is to challenge the assumption on which any such scheme is based, and not to spend time in "constructive criticism" of the details in which its application is expressed. If this criticism is not supplied the blame must fall on the friends of the proposal, not on its opponents.

Another comment invited by THE HOSPITAL article (*loc. cit.*) relates to the phrase "We are not deeply enamoured of the idea of State Medical Service, but we cannot but be aware that the signs of the times point that way." What exactly does this mean? If it implies that public opinion in the

**A State Medical Service—(*continued*).**

profession, or among laymen, has accepted or is certain to accept the principle the proposition must be strongly challenged. Certainly many of the younger men now engaged on the work of the R.A.M.C. have been attracted by a scheme which guarantees an early and secure income, fixed hours of work, regular holidays, and a retiring pension. But the organised opinion of the profession, so far as it has been uttered, has steadily and forcibly expressed itself as entirely opposed to the organisation of a State Medical Service. Whether any considerable degree of lay thought has been applied to the question may be doubted, but certainly some of those who advocate large measures of social reform are not in favour of it. In any event, until the "times" do something more than "point" to so large and serious a proposal it is premature to accept this as inevitable, and an organ of opinion which so readily yields to the "times" would appear to be substituting for its duty of guidance the performance and policy of the jumping cat. There are excellent reasons why Colonel Maurice's scheme should be placed before the profession and the public, but among these the claim, or lament, that a State Medical Service is inevitable most certainly ought not to be included. The sufficient defence of publication is that the scheme helps both friends and opponents to realise, perhaps for the first time, exactly what such a Service would mean when applied in actual practice. It therefore clarifies the position and may so far help discussion. Both because of this and because of the thoughtful care spent over its construction there is ample justification for its appearance, and there is no need to try to strengthen such a position by a cloudy suggestion that the "times point" this way.

There is yet another necessity for comment on The Hospital article of November 30. In the latter part of the article it is suggested that no "proposals and schemes" for a State Medical Service have been advanced from "the rank and file" of the largest of the various medical organisations. The statement is literally true, for the "rank and file" have almost without exception adopted the policy of opposition to any State Medical Service. But it ought in fairness to be recognised that this policy is not a barren negative or what The Hospital calls a "*non-possumus* attitude." On the contrary, it includes a carefully worked-out plan to obtain a maximum of medical service and an enlarged sphere of medical usefulness while at the same time preserving the freedom of judgment and of action of the individual medical practitioner. Colonel Maurice, in the view that a State Medical Service is near at hand, has worked out in admirable fashion a scheme to meet this. But with not less care one which avoids the necessity for such a Service has been prepared and is now before the profession. It is something to know exactly what a State Medical Service means. But it is not the only Richmond in the field.

